# Silicon Alleviates Iron Deficiency in Barley by Enhancing Expression of Strategy II Genes and Metal Redistribution

**DOI:** 10.3389/fpls.2019.00416

**Published:** 2019-04-05

**Authors:** Dragana B. Nikolic, Sofija Nesic, Dragana Bosnic, Ljiljana Kostic, Miroslav Nikolic, Jelena T. Samardzic

**Affiliations:** ^1^Laboratory for Plant Molecular Biology, Institute of Molecular Genetics and Genetic Engineering, University of Belgrade, Belgrade, Serbia; ^2^Plant Nutrition Research Group, Institute for Multidisciplinary Research, University of Belgrade, Belgrade, Serbia

**Keywords:** iron deficiency, silicon, Strategy II Fe acquisition, gene expression, barley (*Hordeum vulgare*), microelement accumulation

## Abstract

The beneficial effects of silicon (Si) have been shown on plants using reduction-based strategy for iron (Fe) acquisition. Here we investigated the influence of Si on Fe deficiency stress alleviation in barley (*Hordeum vulgare*), a crop plant which uses the chelation-based strategy for Fe acquisition. Analyses of chlorophyll content, ROS accumulation, antioxidative status, concentrations of Fe and other micronutrients, along with the expression of Strategy II genes were studied in response to Si supply. Si successfully ameliorated Fe deficiency in barley, diminishing chlorophyll and biomass loss, and improving the activity of antioxidative enzymes, resulting in lowered reactive oxidative species accumulation in the youngest leaves. Alleviation of Fe deficiency stress correlated well with the Si-induced increase of Fe content in the youngest leaves, while it was decreased in root. Moreover, Si nutrition lowered accumulation of other micronutrients in the youngest leaves of Fe deprived plants, by retaining them in the root. On the transcriptional level, Si led to an expedient increase in the expression of genes involved in Strategy II Fe acquisition in roots at the early stage of Fe deficiency stress, while decreasing their expression in a prolonged stress response. Expression of Strategy II genes was remarkably upregulated in the leaves of Si supplied plants. This study broadens the perspective of mechanisms of Si action, providing evidence for ameliorative effects of Si on Strategy II plants, including its influence on accumulation and distribution of microelements, as well as on the expression of the Strategy II genes.

## Introduction

Iron (Fe) is an essential element for all living organisms. Due to its ability to undergo a reversible valence change between ferrous (Fe^2+^) and ferric (Fe^3+^), Fe is involved in vital cellular processes such as photosynthesis and nitrogen fixation. It is a component of numerous proteins and enzymes, including some antioxidative enzymes. However, the same redox properties easily make Fe toxic when present in excess. Free Fe^2+^ ions react directly with O_2_ or catalyze Fenton reaction, leading to oxidative damage; therefore, it must always be in complex with chelators (Broadley, 2012). Although Fe is one of the most abundant elements in Earth’s crust, it is present mostly in the form of ferric hydroxide [Fe(OH)_3_], which is poorly soluble and unavailable to plants (Boukhalfa and Crumbliss, 2002). This problem is emphasized in calcareous soils which make up 30% of the world’s agricultural soils (Guerinot and Yi, 1994). Iron deficiency, hence, is one of the major nutritional concerns, lowering a crop’s yield and nutritional quality. Plants have developed two distinct strategies for iron acquisition from soil (Marschner and Römheld, 1994; Connorton et al., 2017). Dicotyledonous and non-graminaceous monocotyledonous plant species use reduction-based Strategy I, employing ferric chelate reductases (FRO) to reduce Fe(III) to Fe(II) which is then taken up by IRT (Iron Regulated Transporter). Graminaceous monocotyledonous plant species (Poaceae) developed a chelation-based strategy for Fe acquisition (Strategy II). They synthesize metal chelators, mugineic acid (MA) phytosiderophores (PS) and release them into the rhizosphere *via* TOM1 (transporter of mugineic acid family phytosiderophores 1) (Mori, 1999; Nozoye et al., 2011). Iron-phytosiderophore complexes are then taken up by the root cells via yellow stripe1 (YS1) transporters (Curie et al., 2001; Murata et al., 2006; Inoue et al., 2009). Phytosiderophores are synthesized from methionine via S-adenosylmethionine and nicotianamine (NA), in the subsequent steps catalyzed by the enzymes S-adenosyl methionine synthetase (SAMS), nicotianamine synthase (NAS), nicotianamine aminotransferase (NAAT) and deoxymugineic acid synthase (DMAS) (Mori, 1999; Bashir et al., 2006). Genes encoding MA transporters and biosynthetic enzymes are upregulated by Fe deficiency. Phytosiderophores and their precursor non-proteinogenic amino acid NA are also required for both cellular Fe homeostasis and long-distance Fe transport within the plant. While organic acids like citrate are main Fe ligands in apoplast, NA and MAs are essential for Fe chelation and transport in symplast. Moreover, MAs have been identified in the xylem as well as in the phloem, and are considered to be important for long-distance metal transport (Kawai et al., 2001; Nishiyama et al., 2012).

Although not accepted as an essential element, silicon (Si) is well known for its beneficial effects in plants and protection against biotic and abiotic stresses (Hernandez-Apaolaza, 2014; Liang et al., 2015; Pontigo et al., 2015; Coskun et al., 2019); nonetheless the mechanisms of its action are poorly understood. Despite being the second most abundant element in Earth’s crust, intensive crop production without Si fertilizing have reduced the amount of Si in soils available for plants. Therefore, Si fertilizers are being applied increasingly to improve crops’ resistance and yields (Ma and Yamaji, 2006). Recently, an ameliorative effect of Si on micronutrient deficiency has been reported; Si mitigated Fe deficiency symptoms in cucumber, soybean and *Vallerianella locusta* (Gottardi et al., 2012; Gonzalo et al., 2013; Pavlovic et al., 2013). Silicon is considered to enhance metal ion pools in the root and leaf apoplast in conditions of sufficient supply and to intensify its reutilization and transport when plants encounter a lack of the metal micronutrient (Hernandez-Apaolaza, 2014; Liang et al., 2015).

In dicots, Si upregulated the expression of genes involved in the reduction-based strategy for Fe acquisition (Strategy I), as well as genes participating in biosynthesis of Fe-chelating compounds (e.g., organic acids and phenolics), thereby enhancing Fe mobilization from the rhizosphere and reutilization of root apoplastic Fe, as well as xylem translocation of Fe toward the shoot (Pavlovic et al., 2013; Stevic et al., 2016). Moreover, it has been demonstrated that Si increases re-translocation of Fe from old to young leaves associated with upregulated expression of *CsNAS1* and *CsYSL1* as well as increased leaf concentration of NA (Pavlovic et al., 2016).

Si-mediated mitigation of Fe deficiency stress has only been reported for Strategy I plants, and it has even been suggested that Si application has no effect on alleviation of such stress in Strategy II plants (Bityutskii et al., 2010). The distinction between strategy I and strategy II plants is not considered so strict anymore, however, although dicotyledonous plants synthesize phenolic chelating compounds they still require FRO2 and IRT1 to take up iron, and even though functional IRT transporter is found among the Gramineae it is mainly utilized in low oxygen conditions keeping the chelating strategy predominant for Fe entry (Ishimaru et al., 2006; Pedas et al., 2008; Fourcroy et al., 2016). Recently, Gattullo et al. (2016) reported that Fe deficient barley plants promoted mineral weathering, dissolving amorphous mineral phases in the soil and increasing the amount of smectite, by exudation of phytosiderophores. The exudates further drove dissolution of smectite and increased uptake of Si in Fe deficient plants compared to those adequately supplied with Fe.

The objective of the present work was to investigate the ability of Si to ameliorate Fe deficiency stress in a graminaceous plant which uses predominantly the chelation-based strategy for Fe acquisition. Barley (*Hordeum vulgare*) was chosen as a representative plant for this strategy. As we found stress alleviation in Si treated barely, we further analyzed accumulation of Fe and other microelements in leaves, as well as expression of genes involved in Strategy II Fe acquisition, searching for elucidation of the molecular mechanisms underlying the process.

## Materials and Methods

### Plant Materials and Growth Conditions

After soaking in 1 mM CaSO_4_ overnight, seeds of barley (*Hordeum vulgare*, cultivar Rekord; kindly provided by Institute for cereals, Kragujevac, Serbia) were germinated between two sheets of filter paper moistened with saturated CaSO_4_. The 4 days old seedlings were then transferred to a complete nutrient solution containing (in mM): 0.7 K_2_SO_4_, 0.1 KCl, 2.0 Ca(NO_3_)_2_, 0.5 MgSO_4_, 0.1 KH_2_PO_4_, and (in μM): 0.5 MnSO_4_, 0.5 ZnSO_4_, 0.2 CuSO_4_, 0.01(NH_4_)_6_Mo_7_O_24_, 1 H_3_BO_3_. Fe was supplied as NaFe^III^EDTA. Plants were pre-cultured for 4 days in nutrient solution supplied with 20 μM Fe and then grown in either +Fe (80 μM) or in -Fe (Fe-free) nutrient solution, without (-Si) or with (+Si) supply of Si. Si-treatments started after the 4 days preculture, simultaneously with Fe withdrawal. If applied, Si was in the form of Si(OH)_4_ at 1.5 mM. This was freshly prepared by passing Na_2_SiO_3_ (Merck, Sodium silicate solution, extra pure, Fe ≤ 0.005%) through a column filled with cation-exchange resin (Amberlite IR-120, H+ form; Fluka, Buchs SG, Switzerland). Amberlite IR-120 is also capable of removing ionic Fe forms (Schmid and Gerloff, 1961) from Na_2_SiO_3_, thus preventing any possible contamination of the nutrient solution with Fe. The pH of nutrient solutions was adjusted to 6.0 and the nutrient solutions were renewed completely every 2 days and continuously aerated. Plants (15 seedlings per pot of 3L) were grown under controlled environmental conditions with a photoperiod of 16 h:8 h and temperature regime of 25°C:21°C (light:dark), photon flux density of 250 μmol m^-2^ s^-1^ at plant height.

### Chlorophyll Determination

Chlorophyll content in the youngest fully developed leaves was approximated non-destructively using a portable ChlorophyllMeter SPAD-502 device (Minolta Camera Co., Osaka, Japan).

### Determination of Microelements in Plant Tissues

After harvest roots are washed in 1 mM EDTA, and twice in destiled water. Roots and leaves were oven dried at 70°C for 48 h, weighed and hand ground. Dry plant material (0.2 g) was digested in 3 ml concentrated HNO_3_ + 2 ml H_2_O_2_ for 1 h in a microwave oven (Speedwave MWS-3+; Berghof Products + Instruments GmbH, Eningen, Germany). Samples were then transferred into 25 ml plastic flasks and flask volume was adjusted to 25 ml with deionized H_2_O. Fe was determined by inductively coupled plasma optical emission spectroscopy (ICP-OES, SpectroGenesis EOP II; Spectro Analytical Instruments GmbH, Kleve, Germany).

Determination of Si concentration was performed as described in Pavlovic et al. (2013).

### ROS Detection

For fluorescent ROS (reactive oxygen species) detection, the youngest leaves were vacuum infiltrated with dichloro- dihydrofluorescein (H2DCF DA; Sigma-Aldrich) essentially as described in Zhang and Qiu (2007). The leaves were visualized by Olympus BX51 fluorescent microscope with appropriate filters for detection of SpectrumGreen. All images were taken using the same settings (exposure 0.02, bright 166, black 107).

### Assay of Antioxidative Enzymes and Compounds

Leaves were homogenized in 50 mM potassium phosphate buffer (pH 7.0) including 0.1 mM EDTA and 2% (v/v) PVP. The homogenates were centrifuged at 14,000 g for 15 min at 4°C and the supernatants used for enzyme activity assays. The protein content was determined according to Bradford (1976) with bovine serum albumin as a standard.

APX (ascorbate peroxidase, EC 1.11.1.11) activity was determined according to Nakano and Asada (1981) by following the rate of ascorbate oxidation at 290 nm. Reaction mixture contained 50 mM potassium phosphate buffer, pH 7.0, 0.1 mM of EDTA, 0.25 mM ascorbate, 1.27 mM H_2_O_2_ and leaf extract. The enzyme activity was determined using an extinction coefficient of 2.8 mM^-1^ cm^-1^. One unit of APX activity was defined as the amount of enzyme needed for the oxidation of 1 μmol of ascorbate per minute and the specific activity was expressed as units per mg protein.

SOD (Superoxide dismutase, E.C. 1.15.1.1) activity was determined essentially as described by Giannopolitis and Ries (1977) by measuring its ability to inhibit photochemical reduction of nitroblue tetrazolium (NBT) at 560 nm. The reaction mixture contained 50 mM potassium phosphate buffer (pH 7.0), 0.1 mM of EDTA, 75 μM NBT, 13 mM methionine, 2 μM riboflavin and leaf extract. One SOD unit was taken to be the amount of enzyme causing 50% inhibition of NBT reduction and expressed in units per mg protein.

CAT (catalase, E.C.1.11.1.6) activity was determined by following the consumption of H_2_O_2_ at 240 nm according to the method of Aebi (1984). The reaction mixture contained 50 mM potassium phosphate buffer (pH 7.0), 0.1 mM of EDTA, 15 mM H_2_O_2_ and leaf extract. One unit of CAT activity was defined as the amount of enzyme that catalyzes the decomposition of 1 μmol of H_2_O_2_ per minute and the specific activity was expressed as units per mg protein (extinction coefficient 0.0436 mM^-1^ cm^-1^).

Total glutathione concentration was determined essentially as described in Tommasi et al. (2001). Total ascorbate was measured following method described in Vidović et al. (2015).

### RNA Extraction and cDNA Synthesis

Root and leaf tissue samples (0.5–1 g FW) were frozen in liquid N_2_ and ground thoroughly in a mortar. RNA was isolated using the RNeasy^®^ Mini Kit (Qiagen) as described in the RNeasy^®^ Mini Handbook. Before cDNA synthesis DNA was removed from RNA samples using Ambion DNA-free DNase Treatment and Removal Reagents. First strand cDNA was synthesized from 1 μg of RNA with M-MuLV reverse transcriptase (Thermo Scientific) and random hexamer primers (Applied Biosystems, FosterCity, CA, United States) according to the manufacturer’s instructions. The cDNA was diluted 1:5 (v/v) with nuclease-free H_2_O (except when determining target genes expressed at low levels, *HvTOM1* and *HvNAS1* in leaves) and used as a template for Real-time PCR with primers designed for barley.

### Real-Time Quantitative PCR

Real-time PCR reactions were performed in 25 μl volume containing 400 nM of each primer and 1X SYBER Green PCR master mix (Thermo Fisher Scientific), on the ABI Prism 7500 Sequence Detection System (Applied Biosystem), as described in Pavlovic et al. (2013). Actin gene was used as the endogenous control. Primers and accession numbers of housekeeping and target genes are listed in Supplementary Table S1. Level of expression in control plants, grown in optimal Fe supply, was used as a calibrator, i.e., all the expression levels that were normalized to the level of actin gene were subsequently normalized to the expression in the control plants, according to Pfaffl method (Pfaffl, 2001). Gene expression level of plants supplied with Fe was set to 1.0.

### Statistical Analysis

Data were subjected to analysis of variance using the statistical software SPSS for windows and means were compared by Tukey’s test at 5% significance level (*p* < 0.05).

## Results

### Si Alleviates Fe Deficiency Symptoms and Oxidative Stress in Barley

**FIGURE 1:**
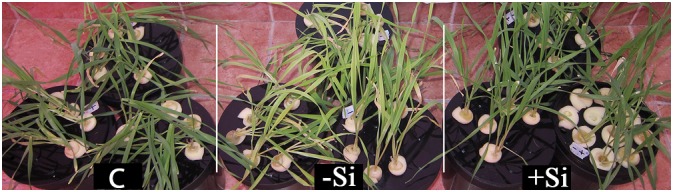
Effect of Si nutrition on visual symptoms of Fe deficiency in barley (*Hordeum vulgare*). Plants were pre-cultured for 4 days in a nutrient solution supplied with 20 μM NaFeIIIEDTA and then grown for 3 weeks in Fe-free nutrient solution, with or without a supply of 1.5 mM Si(OH)_4_ (+Si and -Si, respectively). Control plants (C) were grown in solution supplied with 80 μM NaFeIIIEDTA, without Si.

To examine whether Si alleviates Fe deficiency in Strategy II plants, barley has been chosen as a representative of gramineous plants, which predominantly use the chelating strategy for Fe acquisition. Si successfully mitigated Fe deficiency symptoms in barley pre-cultured for 4 days with 20 μM NaFe^III^EDTA and further grown in hydroponics for 3 weeks without Fe (Figure 1).

Plants grown without Si were severely chlorotic, while in Si-supplied plants chlorosis was alleviated. The effect was the most prominent in the youngest fully expanded leaves. These observations were confirmed by measuring Spectral Plant Analysis Diagnostic (SPAD) index. The relative chlorophyll content was significantly higher in leaves of Si fed plants. Si also promoted the growth of Fe deprived plants. Fresh and dry biomasses of the youngest leaves were considerably higher in Si supplied plants (Table 1).

**Table 1:** Effect of Si nutrition on dry weight (DW) of root, old leaf (L1), and mature leaf (L2) and FW, DW, and chlorophyll content (SPAD units) of the youngest fully expanded leaves of barley.

				Youngest leaf		
Treatments	DW per root (mg)	DW per old leaf (mg)	DW per mature leaf (mg)	FW per leaf (mg)	DW per leaf (mg)	SPAD
C	6.8 ± 1.2 ab	13.3 ± 0.5 a	13.0 ± 1.7 a	219.2 ± 1.7 a	21.3 ± 0.2 a	29.7 ± 2.7 a
-Si	6.6 ± 0.7 b	11.9 ± 0.7 b	12.3 ± 0.6 a	177.5 ± 7.3 c	17.3 ± 0.7 c	10.2 ± 1.5 b
+Si	7.9 ± 0.8 a	13.0 ± 0.3 a	13.0 ± 0.0 a	210.9 ± 4.6 b	20.6 ± 0.4 b	27.5 ± 3.3 a

*Plants were grown as described in the legend of Figure 1 (C- control plants grown optimally supplied with Fe; -Si – plants grown in the absence of Fe and Si; +Si – plants grown in the absence of Fe and supplied with Si). Data shown are means ± s.d. (n = 3). Significant differences (P < 0.05) between treatments are indicated by different letters.*

**FIGURE 2:**
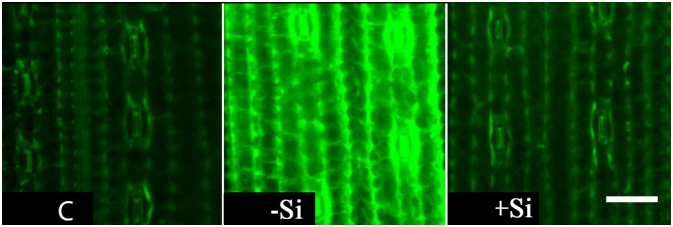
Effect of Si nutrition on ROS accumulation in leaves of iron-deficient barley seedlings. Representative fluorescent microscopy images of the youngest fully expanded leaves showing ROS accumulation as green fluorescence with the probe H_2_DCF DA. The plants were treated as described in the legend of Figure 1 (C- control plants grown optimally supplied with Fe; -Si – plants grown in the absence of Fe and Si; +Si – plants grown in the absence of Fe and supplied with Si). Scale bar = 40 μm.

One of the consequences of Fe deprivation can be oxidative stress due to decreased activity of electron transport chains and reduced activity of some antioxidative enzymes. Therefore, we examined levels of reactive oxygen species (ROS) in the youngest, chlorotic leaves. Intracellular ROS accumulation was monitored using fluorescent dye DCF-DA. An intense fluorescent signal was detected in leaves of iron deprived plants, indicating highly elevated accumulation of ROS. The signal was markedly reduced in leaves of Si treated plants, reversing it to the control level (Figure 2).

Since ROS accumulation observed in leaves of iron-deficient plants may be, in part, a result of the reduced activity of antioxidative enzymes, we measured the activity of ascorbate peroxidase, catalase and superoxide dismutase (Table 2). As previously reported for different plant species (Ranieri et al., 2001; Zaharieva et al., 2004; Sun et al., 2007; Ramírez et al., 2013) iron deficiency caused a huge decrease in the activity of the heme-containing enzymes ascorbate peroxidase and catalase in barley leaves. Silicon supply remarkably improved the activities of both enzymes, restoring them to the levels found under iron-sufficient conditions. On the other hand, aside from the Fe isoform, SOD also comprises Mn and Cu-Zn isoforms and we did not detect any significant change in its activity among the treatments. The non-enzymatic antioxidants ascorbate and glutathione are also involved in keeping the cellular redox homeostasis and their levels are found to be altered during Fe deprivation (Zaharieva et al., 2004; Ramírez et al., 2013). We detected a significant increase in glutathione content in the youngest leaves under Fe deficiency, irrespective of Si supply. The level of ascorbate did not change significantly among the treatments (Table 2).

**Table 2:** Effect of Si supply on activity of antioxidative enzymes and concentration of non-enzymatic antioxidants in the youngest fully expanded leaves.

Treatment	APX	CAT	SOD	GSH	Asc
C	0.120 ± 0.048 a	171 ± 29 a	134 ± 16 a	156 ± 10 b	2043 ± 205 a
-Si	0.051 ± 0.012 b	124 ± 27 b	133 ± 4 a	201 ± 9 a	2165 ± 185 a
+Si	0.143 ± 0.022 a	177 ± 10 a	124 ± 10 a	200 ± 12 a	2261 ± 228 a

*Enzyme activities are given as u/mg protein; GSH and Asc in nmol g^-1^ FW. The plants were treated as described in the legend of Figure 1 (C- control plants grown optimally supplied with Fe; -Si – plants grown in the absence of Fe and Si; +Si – plants grown in the absence of Fe and supplied with Si). Data shown are means ± s.d. (n = 3). APX, Ascorbate peroxidase; CAT, catalase; SOD, superoxide dismutase; GSH, glutathione; Asc, ascorbate. Significant differences (P < 0.05) between treatments are indicated by different letters.*

**Table 3:** Concentration of microelements and Si in the youngest leaves of barley, 3 weeks after Fe withdrawal.

	Microelement concentration in the youngest leaves (μg g^-1^ DW)				
Treatment	Fe	Mn	Zn	Cu	Si (% DW)
C	104.1 ± 31.7 a	54.0 ± 13.0 c	75.1 ± 27.8 b	11.3 ± 3.7 c	0.61 ± 0.09 b
-Si	37.9 ± 8.2 c	154.0 ± 16.6 a	188.2 ± 25.0 a	31.2 ± 5.6 a	0.40 ± 0.14 b
+Si	64.5 ± 11.5 b	100.1 ± 9.6 b	114.6 ± 15.2 b	22.1 ± 5.7 b	1.26 ± 0.11 a

*The plants were treated as described in the legend of Figure 1 (C- control plants grown optimally supplied with Fe; -Si – plants grown in the absence of Fe and Si; +Si – plants grown in the absence of Fe and supplied with Si). Data shown are means ± s.d. (n ≥ 3). Significant differences (P < 0.05) between treatments are indicated by different letters.*

### Si Increases Total Fe Concentration andContent and Modulates Concentrationand Content of Some OtherMicroelements in the Youngest Leavesof Fe Deficient Barley

**FIGURE 3:**
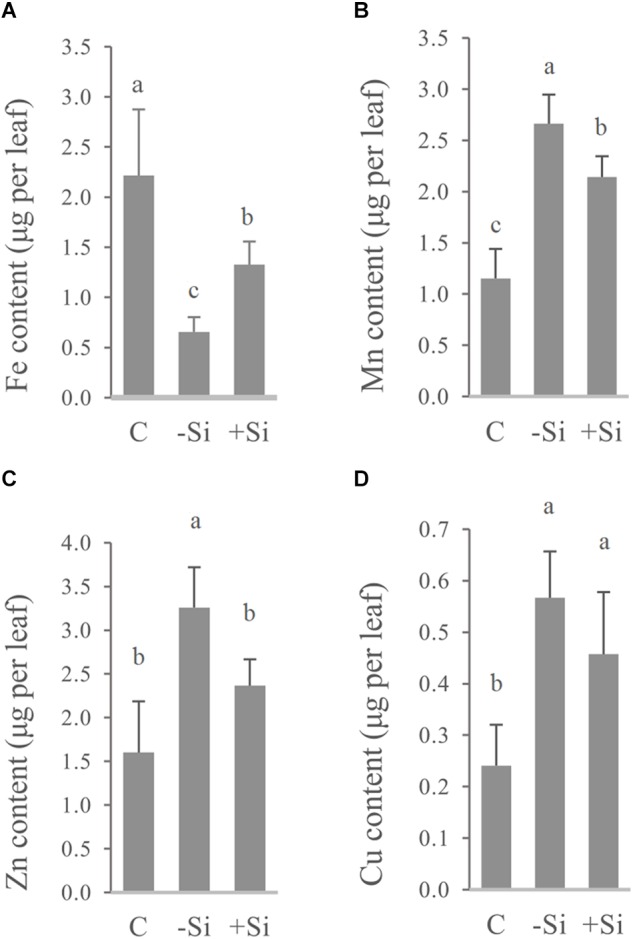
Effect of Si nutrition on micronutrient content in the youngest fully developed barley leaves (third leaves, L3), 3 weeks after Fe withdrawal. Total leaf content of **(A)** Fe, **(B)** Mn, **(C)** Zn, and **(D)** Cu. The plants were treated as described in the legend of Figure 1 (C- control plants grown optimally supplied with Fe; -Si – plants grown in the absence of Fe and Si; +Si – plants grown in the absence of Fe and supplied with Si). Data shown are means ± s.d. (*n* ≥ 3). Significant differences (*P* < 0.05) between treatments are indicated by different letters.

Increased chlorophyll content as well as APX and CAT activities under Si supply, indicated increased iron concentration and content in the youngest leaves. Along with Fe we also measured the leaf content and concentration of Mn, Zn, and Cu, to obtain information for other elements that could be perturbed during iron deficiency. As we have expected, Si significantly raised Fe concentration and content in the youngest leaves of barley (Table 3 and Figure 3A).

On the other hand, concentrations of Mn, Zn, and Cu were increased in Fe deficient leaves (Table 3), and were above critical levels of toxicity according to Pedas et al. (2014) and Ryzhenko et al. (2016). However, concentrations of all three elements, as well as leaf contents of Mn and Zn in Si-treated Fe deprived plants were significantly reduced, implying the additional protective role of Si (Table 3 and Figure 3). Increased concentration of Si was also shown in the youngest leaves of Si treated plants (Table 3).

**FIGURE 4:**
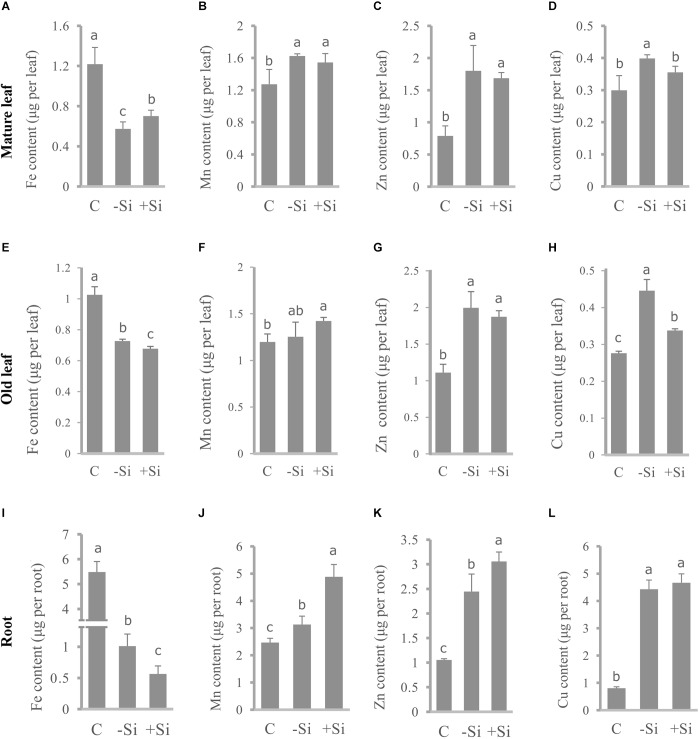
Effect of Si nutrition on micronutrient content in different organs of barley, 3 weeks after Fe withdrawal. Mature leaf (second leaf, L2): **(A–D)**. Old leaf (first leaf, L1): **(E–H)**. Root: **(I–L)**. Content of Fe: **(A,E,I).** Content of Mn: **(B,F,J)**. Content of Zn: **(C,G,K)**. Content of Cu: **(D,H,L)**. The plants were treated as described in the legend of Figure 1 (C- control plants grown optimally supplied with Fe; -Si – plants grown in the absence of Fe and Si; +Si – plants grown in the absence of Fe and supplied with Si). Data shown are means ± s.d. (*n* = 3). Significant differences (*P* < 0.05) between treatments are indicated by different letters.

**FIGURE 5:**
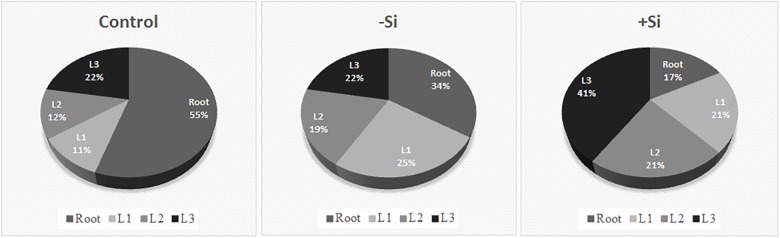
Relative distribution of Fe content in different plant organs. Plants were treated as described in Figure 1. Leaf positions (from the base to the youngest leaf): L1, L2, L3.

To determine Fe distribution in plant organs during the Fe deprivation we measured Fe content in roots and three leaf maturity stages (L1–L3) (Figure 4, 5). More than half of Fe in control plant is deposited in roots; in leaves the majority of Fe is present in the youngest ones. Under Fe deficiency only one third of Fe is present in roots and distribution of Fe among leaves is also changed, the highest content is determined in the oldest leaves. In Si supplied plants, grown without Fe, Fe content in roots decreased even more; only 17% of the total Fe in plant was retained in this organ. The youngest leaves of silicon fed plants were the most abundant in Fe content.

In parallel, we also measured contents of other essential metals, such as Mn, Zn, and Cu and investigated differences in their distribution under Fe deficiency and upon Si supply (Figure 4). All the metals showed similar pattern of the distribution between organs in Fe deficient plants – increase of the content in the roots and leaves compared with the control, with the exception of Mn in L1 and L2 which were equal to the control plants. Si nutrition additionally increased the metal content in roots (except Cu). Significant decrease in Cu content, under influence of Si, was detected in old and mature leaves (L1 and L2), in contrast to Mn and Zn that were less present only in the youngest leaves. Mn and Zn contents were almost unchanged in L1 and L2, in respect to Si supply. Concentrations of Fe, Mn, Zn, and Cu in root, old and mature leaf are given in a Supplementary Table S2.

### Si Accelerates Increase in Expression of Genes Related to Strategy II Fe Acquisition in Roots and Upregulates the Gene Expression in Leaves of Fe Deficient Barley

Previous studies have shown modulation of the expression of genes involved in Strategy I Fe acquisition and Fe re-translocation upon Si treatment (Pavlovic et al., 2013, 2016). In this work, we monitored changes in the expression of genes participating in Strategy II Fe acquisition: *HvDMAS1*, *HvTOM1*, and *HvYS1*, responsible for phytosiderophore DMA biosynthesis and release, and metal-DMA complex uptake, respectively. We also analyzed the expression of *HvNAS1*, the gene for nicotianamine synthase, involved in the biosynthesis pathway of phytosiderophores.

**FIGURE 6:**
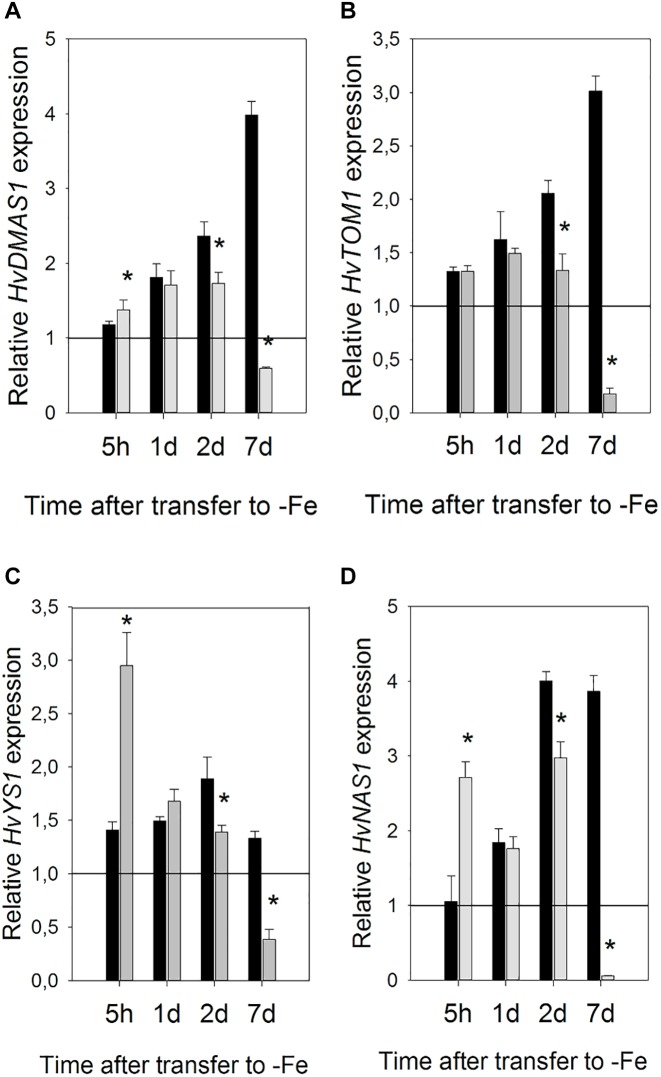
Effect of Si supply on the relative expression level of **(A)** *HvDMAS*, **(B)** *HvTOM*, **(C)** *HvYS1* and **(D)** *HvNAS1* in barley roots 5 h and 1, 2, and 7 days after iron withdrawal from the nutrient solution. The plants were treated as described in the legend of Figure 1 (-Si – plants grown in the absence of Fe and Si; +Si – plants grown in the absence of Fe and supplied with Si). Gene expression is normalized to the values obtained for control plants grown in optimal Fe supply, which was set to 1.0. Black bars -Si; gray bars +Si. Data shown are means ± s.d. (*n* = 3). Significant differences (*P* < 0.05) between -Si and +Si treatments for each time point are indicated by asterisks.

When compared to the gene expression level in the control plants (which was shown set to 1, on the graph in Figure 6, as it was used as the calibrator), the expression of all the examined genes was unchanged or slightly increased in Fe-deficient roots, 5 h after Fe withdrawal. In Si treated roots, however, when compared to both the control and Fe-deficient plants, the expression level of all genes, except *HvTOM1*, was instantly and significantly elevated 5 h after Fe withdrawal. Si-dependent acceleration of gene expression response is most evident for *HvYS1* and *HvNAS1* genes, which were increased more than twofold in +Si roots in comparison to -Si roots. On the first and second day, the expression of all examined genes was higher from the control level in both -Si and +Si roots, however, on the second day, the expression in Si supplied roots was decreased compared to -Si roots. The biggest differences in the transcript levels between treatments were observed on the seventh day. While in the -Si roots the expression of all the genes was kept on the same high level as before (*HvYS1* and *HvNAS1*) or raised even higher (*HvDMAS1* and *HvTOM1*), in Si supplied roots the expression of all genes decreased greatly, even compared to the control (Figure 6).

**FIGURE 7:**
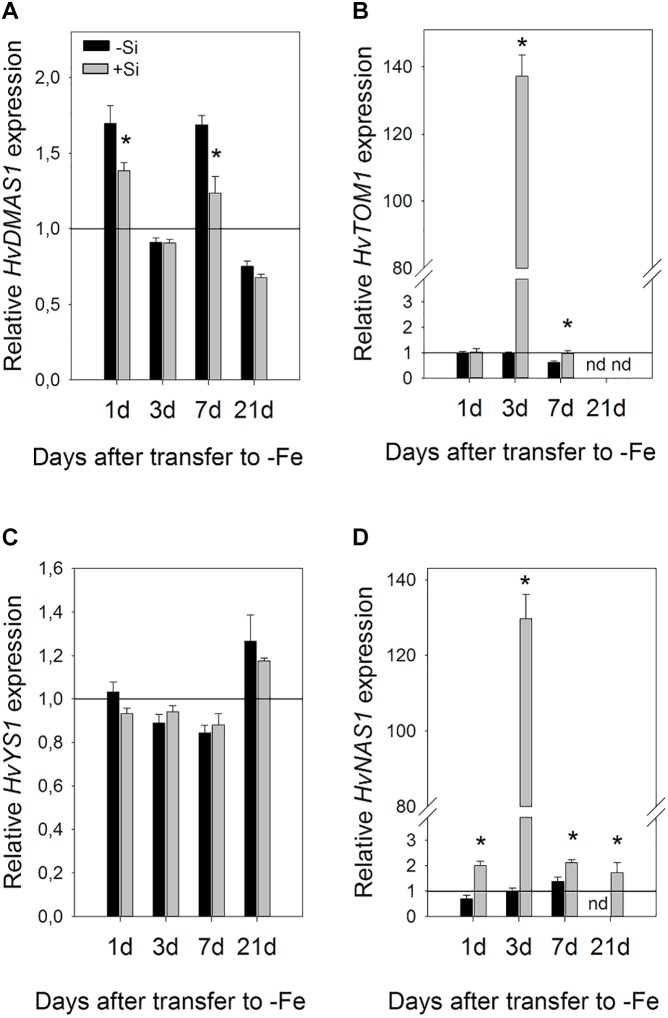
Effect of Si supply on the relative expression level of **(A)** *HvDMAS*, **(B)** *HvTOM*, **(C)** *HvYS1*, and **(D)** *HvNAS1* in the barley leaves 1, 3, 7, and 21 days after iron withdrawal from the nutrient solution. The plants were treated as described in the legend of Figure 1 (-Si – plants grown in the absence of Fe and Si; +Si – plants grown in the absence of Fe and supplied with Si). Gene expression is normalized to the values obtained for control plants grown in optimal Fe supply, which was set to 1.0. Black bars -Si; gray bars +Si. Data shown are means ± s.d. (*n* = 3). Significant differences (*P* < 0.05) between -Si and +Si treatments for each time point are indicated by asterisks.

Si also influenced gene expression in the leaves, however, it was regulated differently compared to the roots (Figure 7). Changes in expression were slower and the greatest influence of Si was seen on the third day. Si remarkably increased expression of *HvNAS1* and *HvTOM1* genes on the third day of Fe deprivation, 134- and 137-fold, respectively. *HvNAS1* was also upregulated on the first and seventh day. *HvTOM1* gene expression was not influenced by Si on the first day, and a small upregulation was detected on the seventh day. On the other hand, no significant change in transcript level of *HvYS1* was detected at any of the tested time points, while *HvDMAS1* expression slightly decreased or remained unaffected.

## Discussion

Recent publications have reported an ameliorative effect of silicon on iron deficient Strategy I plants, in particular cucumber, pumpkin and soybean (Bityutskii et al., 2010; Gonzalo et al., 2013; Pavlovic et al., 2013). Si increased the apoplastic Fe pool in cucumber roots and enhanced Fe acquisition and translocation toward apical shoot parts by the accumulation of Fe-mobilizing compounds and acceleration and enhancement of the expression response of the relevant genes (Pavlovic et al., 2013; Bityutskii et al., 2014). To date, beneficial effects of Si on Fe deficiency stress in Strategy II plants have not been reported; moreover, it has been suggested that Si cannot alleviate stress in the chelation-based strategy (Bityutskii et al., 2010). In the present study we have clearly demonstrated an ameliorative effect of Si on Fe deficiency stress in barley, a plant with predominant Strategy II Fe acquisition and shown Si-induced modulation of Strategy II genes expression.

The most obvious effect of Si was successfully prevented chlorosis and biomass reduction of the youngest leaves (Table 1). One of the consequences of iron deprivation can also be an imbalance of cellular redox homeostasis. We have shown a high intracellular accumulation of ROS in the youngest leaves of Fe deficient barley, as detected by DCF-DA fluorescent dye, and a decrease in ROS level in the presence of Si (Figure 2). ROS may serve as signaling molecules, which trigger stress responses to various abiotic and biotic stresses, but their accumulation could also lead to oxidative damage of cellular components (Mittler et al., 2004). These dual effects have also been observed, although not completely understood, in iron deficiency stress. Prolonged Fe deficiency can result in the accumulation of ROS in leaves, due to lowered activity of Fe-dependent antioxidative enzymes and perturbances in electron transport chains in mitochondria and chloroplasts (Ranieri et al., 2001; Sun et al., 2007; Ramírez et al., 2013). ROS accumulation in Fe deprived leaves, and a restoration of basal ROS level in the youngest leaves of Si-supplied barley (Figure 2) was associated with changes in antioxidative enzyme activities. APX and CAT activities were significantly decreased in Fe deficient plants, but recovered upon Si treatment, while SOD activity was not significantly changed (Table 2). APX and CAT are heme-containing enzymes and their activities are strongly dependent on Fe status, while SOD exists in three isoforms containing Fe, Mn, or Cu/Zn, allowing maintenance of enzyme activity in respect to metal availability. Important players in maintaining cellular redox homeostasis are components of the ascorbate – glutathione cycle. Among other functions, ASC and GSH are found to protect *Arabidopsis* from iron deficiency, preserving redox homeostasis and improving internal iron availability (Zaharieva et al., 2004; Ramírez et al., 2013). However, even though we found an increased GSH level in barley leaves as a response to Fe deprivation, the influence of Si was not detected. ASC content was not altered among the treatments (Table 2).

As we assumed, silicon significantly increased the total iron content in the youngest leaves (Figure 3A), which was in accordance with a lower ROS level, elevated APX and CAT activities and chlorophyll level. It has already been shown that Si accelerates utilization of Fe from root apoplast in cucumber (Pavlovic et al., 2013). However, unlike the cucumber where the older leaves are the source of iron that is used for its Si-induced remobilization to younger leaves, we have found that in the barley the older leaves are of minor importance, with the main source of iron actually being the root.

The Fe pool translocated from root is much greater in Si supplied plants, compared to -Si plants, which constitutes a sufficient supply of Fe for the youngest leaves. It is also important to note that even though the root Fe content and concentration were depleted, roots of Si-fed plants appear to be superior in sustaining the stress, having higher biomass (DW) compared to roots of both -Si and control plants. It would suggest that Si does not stimulate remobilization of Fe from vital compartments, but rather from the deposits such as apoplast and root surface, which otherwise stay unused in -Si plants. In rice, the high resolution images, using SEM-EDX and LA-ICP-MS techniques, revealed that under Fe deficiency, Si supply has significantly decreased the Fe concentration in epidermal cells and root surface, but has, on the other hand, increased its concentration in the endodermal and vascular cylinder cells (Carrasco-Gil et al., 2018). The authors have suggested that the Fe plaque formed on root surface, during the Fe sufficient period, could function as an Fe source when plants need it.

The enhanced expression of the Fe acquisition genes in the early phase of the stress, as well as the greatly increased expression of the genes involved in phytosiderophores biosynthesis and transport in leaves, may have additively contributed to the efficient translocation of Fe from root to shoot.

Si led to an expedient increase in the expression of genes involved in Strategy II Fe acquisition in roots at the early stage of Fe deficiency stress. NA synthase and genes involved in phytosiderophore DMA synthesis and uptake of Fe-DMA complexes (*HvDMAS* and *HvYS1*) were induced by Si as soon as 5 h after withdrawal of Fe from the nutrient medium, while in the absence of Si a rise in their expression was delayed 1 or 2 days (Figure 6). Acceleration of gene expression response has previously been shown for Strategy I Fe acquisition genes *FRO2*, *IRT1*, and *HA1*, however the response was slower compared to the response of Strategy II genes examined in this study. Si-induced enhancement of their expression was greatest 1 and 3 days after Fe withdrawal (Pavlovic et al., 2013).

A dramatic increase in the expression of *HvNAS1* and *HvTOM1* genes in the leaves 3 days after Fe withdrawal (Figure 7) could play an important role in utilization and redistribution of iron within the shoot. *NAS1* encodes the enzyme responsible for biosynthesis of nicotianamine (NA), an important Fe ligand involved in Fe transport through phloem as well as in intracellular Fe chelation and short distance transport (Scholz et al., 1992; Hell and Stephan, 2003). Moreover, NA is also a biosynthetic precursor of deoxymugineic acid. *HvTOM1* is a phytosiderophore efflux transporter. The *TOM1* gene from rice was upregulated in both roots and leaves under Fe deficiency, and its promoter activity was detected in leaf phloem. *Xenopus laevis* oocytes expressing *TOM1* or *HvTOM1* were found to release DMA but not NA (Nozoye et al., 2011). It is possible that elevated expression of *HvNAS1* under the influence of Si, is necessary as an important step in DMA biosynthesis, and that phytosiderophores are crucial not only for Fe acquisition from the rhizosphere, but also in Fe utilization and re-translocation in the shoots of graminaceous plants. The importance of DMA in plant internal metal transport was suggested by chemical speciation of iron-binding ligands that revealed DMA as a dominant chelator in rice phloem sap (Nishiyama et al., 2012). Phytosiderophores were also detected in the xylem sap of rice and barley plants (Mori et al., 1991; Kawai et al., 2001).

Although we have not detected changes in *HvDMAS1* and *HvYS1* transcript levels in leaves, we cannot exclude the possibility of regulation of their expression on the post-transcriptional level. Besides, other members of the *YSL* gene family may also be influenced by Si.

Dynamics of the changes in gene expression suggest that processes important for Si-enhanced remobilization of Fe from root apoplast and translocation from root to shoot could occur mostly in the early phase of the stress response: in root already 5 h after Fe removal and in shoot around the third day. It is possible that the pool of Fe is firstly translocated from roots to the first leaves which are the only ones expanded by the 7th day, and that subsequently the pool is re-mobilized to the younger developing leaves.

It is also possible that Fe is translocated from root during expansion of the second and the third leaf, too, and that the pool of Fe is directly transferred from root to the leaf that is the youngest and developing in at that moment. Direct translocation of Fe from roots to young leaves via phloem, in graminaceous plants, have been indicated by the visualization of the real-time translocation of iron (Fe) in barley using a positron-emitting tracer imaging system (PETIS) (Tsukamoto et al., 2009). We have observed huge substantial changes only in HvTOM1 and HvNAS1 expression on the 3rd day, and smaller differences between treatments on the 7th and 21st, however, we have no insight into the fluctuation of the gene expression and production of phytosiderophores during the whole treatment and so that we cannot exclude the possibility that they fluctuate dynamically in order to support remobilization of Fe to developing leaves when it is needed the most.

Iron deficiency can perturb the homeostasis of some other essential elements. Increased concentrations of Mn, Co, Zn, and Cd were found in A. thaliana under iron deficiency (Baxter et al., 2008). Le et al. (2016) suggested that down-regulation of the Fe acquisition machinery in prolonged Fe deprivation is required in order to prevent damage from the accumulation of other metals. As shown in Table 3 and Figure 3, Si significantly decreased Mn, Zn, and Cu concentrations and Mn and Zn content in the youngest fully expanded leaves, contrary to its effect on Fe content. The reduced accumulation of Mn and Zn may have also contributed to the Si-induced lowering of ROS level. It is noteworthy that expression of all the examined genes remained elevated in roots even in prolonged iron deficiency in the absence of Si, while Si supply decreased greatly their expression on the seventh day of treatment (Figure 6). Fe acquisition machinery can only have positive effects on Fe uptake from root apoplast in the early stages of Fe deprivation, but in a longer period of total absence of Fe it cannot further improve Fe status. In our opinion, faster Fe mobilization in the root and its improved availability in leaves reduced Fe deficiency stress in Si treated plants, which resulted in decreased expression of Fe acquisition genes during prolonged treatment. Lowered expression of Strategy II genes in the prolonged stress may have led to the reduced uptake of some metal ions. However, the increased content of Mn and Zn in the root, while being lowered in the youngest leaves, may suggest that Si protects young, developing plant organs by retaining the potentially harmful metals in the root. Further investigation, employing imaging techniques, may give us information about localization and distribution of different metals within the root and contribute to elucidation of the mechanism of Si-induced metal deposition in root.

## Conclusion

**FIGURE 8:**
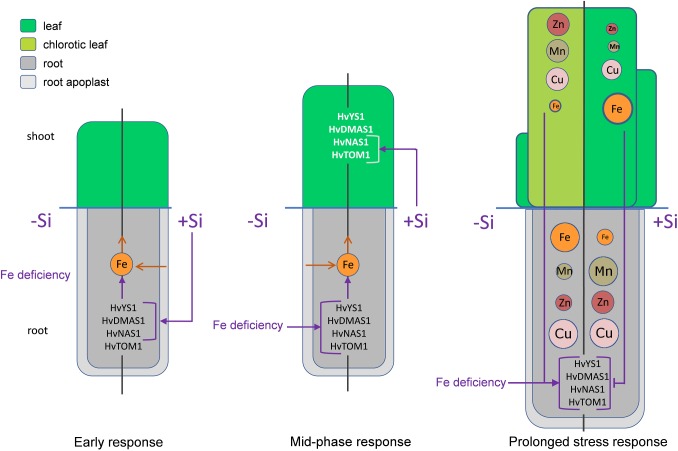
The proposed model of Si-mediated alleviation of Fe deficiency in barley. In the early phase (5 h after Fe withdrawal) Si stimulates the expression of Strategy II genes in root, enhancing that way Fe uptake from the root apoplast and its remobilization toward shoot. In the mid-phase response (2 and 3 days after Fe withdrawal) the expression of Strategy II genes in root starts to increase under the influence of Fe deficiency. In this phase, Si enhances the expression of *HvTOM1* and *HvNAS1* in the leaf. As a consequence, after prolonged period of Fe deficiency, the status of Fe in the youngest leaf of Si treated plants is improved compared to the low Fe concentration in the plants grown without Si. Low Fe status in leaf of -Si plants is a signal for further increase in the gene expression in the root. On the contrary, improved Fe status in Si treated plants inhibits the expression of Strategy II genes. Another consequence of the reduced leaf Fe concentration is an increased uptake of other metals, which leads to elevated concentrations of Zn, Mn, and Cu in shoot. Si treatment increases concentrations and contents of Mn and Zn in root, resulting in decreased translocation toward shoot and lowered accumulation of these metals in the youngest leaves. The left halves of the schemes represent plants grown in the absence of Si, the right halves represent plants supplied with Si. Purple arrows – positive influence, purple T-end lines – inhibitory influence, brown open arrows – flow of the metal ions. Metal ions are represented by circles; the size of the circles in the youngest leaf corresponds to the concentration of the element.

In conclusion, an ameliorative effect of Si on Fe deficiency in barley, a gramineous plant, was shown. Si improved Fe translocation from root to shoot, resulting in an increased Fe content in the youngest leaves. Another protective effect of Si during Fe deficiency stress was decreased accumulation of Mn and Zn in the youngest leaves, by retaining them in root. The influence of Si on the genes involved in Strategy II Fe acquisition is presented for the first time, indicating a role of phytosiderophores in Si-induced stress alleviation. The expedient increase in the expression of Strategy II iron acquisition genes and *HvNAS1* in roots and strongly enhanced *HvTOM1* and *HvNAS1* expression in leaves, was observed in Si supplied plants. We propose a mechanism of Si protective activity in Fe deficient barley and its influence on Strategy II genes (Figure 8). Fe deficiency leads to low yield and plant fitness, but also to the reduced nutritional value of cereal crops widely used for human and animal consumption. In that light, formulation of new eco-friendly Si-based fertilizers might be an important approach to surmount these shortcomings.

## Author Contributions

DN designed and performed the experiments, analyzed data, and wrote the manuscript. SN performed the experiments and analyzed data. DB contributed to the experiments. LK performed ICP-OES measurements. MN and JS initiated the project, analyzed, and discussed the data.

## Conflict of Interest Statement

The authors declare that the research was conducted in the absence of any commercial or financial relationships that could be construed as a potential conflict of interest.
