# Supplementary material for: Silicon Alleviates Iron Deficiency in Barley by Enhancing Expression of Strategy II Genes and Metal Redistribution
Source: Front Plant Sci. 2019 Apr 5;10:416. doi: 10.3389/fpls.2019.00416 (PMC6460936; doi:10.3389/fpls.2019.00416)
Supplement: Supplementary file 1 [file Table_1.doc]

| **gene** | **NCBI GenBank**  **Accession no.** | **primers** |
| --- | --- | --- |
| *Hordeum vulgare* actin | [AY145451.1](https://www.ncbi.nlm.nih.gov/nucleotide/AY145451?report=genbank&log$=nuclalign&blast_rank=3&RID=9FYFP4FU01R) | HvAct-F ccaggtatcgctgaccgtat  HvAct-R gctgagtgaggctaggatgg |
| *Hordeum vulgare* *HvNAS1,* nicotianamine synthase 1 | [AB010086.1](https://www.ncbi.nlm.nih.gov/nucleotide/AB010086?report=genbank&log$=nuclalign&blast_rank=3&RID=9FYW3P6E013) | HvNAS1-F agagctggccttttgattga  HvNAS1-R aaggccggaagcataactct |
| *Hordeum vulgare* *HvYS1*, iron-phytosiderophore transporter | [AB214183.1](https://www.ncbi.nlm.nih.gov/nucleotide/AB214183?report=genbank&log$=nuclalign&blast_rank=1&RID=9FZ482MC013) | HvYS1-F gctttgatatgtggggatgg  HvYS1-R cagcaaggcacaactatgga |
| *Hordeum vulgare* *HvTOM1*, DMA efflux transporter | [AB683951.1](https://www.ncbi.nlm.nih.gov/nucleotide/AB683951?report=genbank&log$=nuclalign&blast_rank=1&RID=9FZE7TBJ013) | HvTOM1-F gcttcaggtgctggtcttct  HvTOM1-R gtccaagtgtcgtcccagat |
| *Hordeum vulgare* *HvDMAS1*, deoxymugineic acid synthase1 | [AB269907.1](https://www.ncbi.nlm.nih.gov/nucleotide/AB269907?report=genbank&log$=nuclalign&blast_rank=2&RID=9FZM3GFW013) | HvDMAS1-F ggactgcctcatcgtcaaga  HvDMAS1-R gacttgtaaggcccgtggtc |

Table S1 - Gene accession numbers and list of primers used in this research.
