# Supplementary material for: Silicon Alleviates Iron Deficiency in Barley by Enhancing Expression of Strategy II Genes and Metal Redistribution
Source: Front Plant Sci. 2019 Apr 5;10:416. doi: 10.3389/fpls.2019.00416 (PMC6460936; doi:10.3389/fpls.2019.00416)
Supplement: Supplementary file 2 [file Table_2.DOC]

**Table S2** Concentration of microelements in root and old and mature leaves of barley, three weeks after Fe withdrawal. The plants were treated as described in the legend of Fig. 1 (C- control plants grown optimally supplied with Fe; -Si – plants grown in the absence of Fe and Si; +Si – plants grown in the absence of Fe and supplied with Si). Data shown are means ± s.d. (n = 3). Significant differences (P < 0.05) between treatments are indicated by different letters.

|  |  | Microelement concentration in roots, old and mature leaves (µg g-1 DW) | | | |
| --- | --- | --- | --- | --- | --- |
|  | Treatment | Fe | Mn | Zn | Cu |
| Root | C | 813.6 ± 62.7 a | 366.1 ± 23.4 c | 156.3 ± 3.8 b | 119.3 ± 7.9 c |
|  | -Si | 154.1 ± 29.6 b | 477.7 ± 46.8 b | 373.2 ± 54.1 a | 675.5 ± 51.3 a |
|  | +Si | 71.5 ± 16.3 c | 618.8 ± 57.4 a | 387.5 ± 24.4 a | 591.7 ± 41.0 b |
| Old leaf | C | 77.4 ± 1.0 a | 88.6 ± 2.4 b | 84.1 ± 11.1 c | 20.5 ± 1.5 c |
|  | -Si | 61.1 ± 4.6 b | 108.7 ± 10.7 a | 166.8 ± 12.8 a | 37.4 ± 3.0 a |
|  | +Si | 52.2 ± 0.5 c | 108.2 ± 0.9 a | 142.5 ± 9.9 b | 26.1 ± 0.9 b |
| Mature leaf | C | 93.8 ± 3.7 a | 98.0 ± 3.2 b | 60.4 ± 4.8 b | 23.0 ± 0.7 c |
|  | -Si | 46.4 ± 3.4 c | 131.8 ± 6.9 a | 145.3 ± 24.5 a | 32.3 ± 1.5 a |
|  | +Si | 53.9 ± 4.5 b | 118.8 ± 8.5 a | 129.6 ± 6.9 a | 27.3 ± 1.5 b |
